# Seroepidemiology of HBV Infection in South-East of Iran; A Population Based Study

**Published:** 2012-05-30

**Authors:** M Salehi, S M Alavian, S V Tabatabaei, Sh Izadi, E Sanei Moghaddam, S Amini Kafi-abad, A Gharehbaghian, S Khosravi, H Abolghasemi

**Affiliations:** 1 Research Center for Infectious Disease and Tropical Medicine, Zahedan University of Medical Sciences, Zahedan, Iran; 2 Baqiyatallah Research Center for Gastroenterology and Liver Disease, Baqiyatallah University of Medical Sciences, Tehran, Iran,; 3 Westfälische Wilhelms-Universität Münster, St.-Marien-Hospital GmbH, Gastroenterologishe Klinik, Lünen, Germany; 4 Zahedan University of Medical Sciences, School of Public Health, Zahedan, Iran; 5 Iranian Blood Transfusion Organization Research Center, Zahedan, Iran; 6 Iranian Blood Transfusion Organization Research Center, Tehran, Iran

**Keywords:** Epidemiology, Hepatitis, HBV, Iran

## Abstract

**Background:**

Hepatitis B virus (HBV) infection is a major risk factor of cirrhosis and hepatocellular carcinoma affecting billions of people globally. Since information on its prevalence in general population is mandatory for formulating effective policies, this population based serological survey was conducted in Sistan and Baluchistan, where no previous epidemiological data were available.

**Methods:**

Using random cluster sampling 3989 healthy subjects were selected from 9 districts of Sistan and Baluchistan Province in southeastern Iran. The subjects’ age ranged from 6 to 65 years old. Serum samples were tested for HBcAb, HBsAg. Screening tests were carried out by the third generation of ELISA. Various risk factors were recorded and multivariate analysis was performed.

**Results:**

The prevalence of HBsAg and HBcAb in Sistan and Baluchistan was 3.38% (95% CI 2.85; 3.98) and 23.58% (95% CI 22.29; 24.93) respectively. We found 8 cases of positive anti-HDV antibody. Predictors of HBsAg or HBcAb in multivariate analysis were age, marital status and addiction.

**Conclusion:**

The rate of HBV infection in Sistan and Baluchistan was higher than other parts of Iran. Approximately 25% of general population in this province had previous exposure to HBV and 3% were HBsAg carriers. Intrafamilial and addiction were major routes of HBV transmission in this province.

## Introduction

Hepatitis B virus (HBV) infection is a major cause of liver disease in the world. WHO has estimated that currently two billions people were infected with HBV and in more than 350 millions people, acute infection has turned to chronic infection. It is also estimated that more than 500,000 deaths occur as a consequence of cirrhosis and hepatocellular carcinoma caused by chronic HBV infection annually.[1][2] One study has showed that 51 to 56% of Iranian cirrhotic patients are hepatitis B surface antigen (HBsAg) positive.[3][4] In Iran, epidemiology of HBV infection has changed dramatically during the last two decades. Seroprevalence of HBsAg has changed from 2.5-7% in 1980s to 1.07-5% in 1990s and to 1-2% in 2000s.[1][5][6][7][8][9][10][11][12][13] Extensive people's knowledge about HBV risk factors, national vaccination program since 1993 for all neonates, vaccination of high risk groups such as healthcare workers and the introduction of disposable syringes for use in vaccinations, hospitals and clinics might justify this decrease.[14]

In 2008, in a systematic review of literature, we gathered and pooled available data on seroepidemiology of HBV infection in general population of Iran. According to our findings, the data were available for only 7 out of 30 provinces of Iran. The HBV infection prevalence in Iran was estimated 2.14% (95% CI: 1.92; 2.35).[15] Despite some problems in the methodology of this meta-analysis, its comprehensive literature review showed that there was a significant lack of data regarding seroepidemiology of HBV infection in Iran. Sistan and Baluchistan is a province with missing data regarding seroepidemiology and distribution of risk factors of HBV infection. From public health view, In addition to seroepidemiology, the distribution of risk factors of HBV infection seems important, since with proper intervention, HBV seroepidemiology can be contained. The major known risk factors for transmission of HBV are HBsAg positive pregnancy, transfusion, hospitalization, tattoo, and intravenous drug abuse and high risk sexual behaviors.[16][17] The lack of information on HBV prevalence and distribution of risk factors among the general population is an obstacle for formulating effective policies to reduce the burden of HBV infection; therefore, this population based study was designed to determinate accurate estimate of HBV infection epidemiology and associated risk factors in Sistan and Baluchistan Province.

Sistan and Baluchistan has 187,502 km^2^ area and 240,574 population which is located at south east of Iran. This province is of great importance as a result of a long common border with Pakistan and Afghanistan that are hyperendemic area regarding HBV infection.[18]

## Materials and Methods

The general population of Sistan and Baluchistan in the southeast of Iran was enrolled. Subjects between 6 and 65 years of age were included. Temporal inhabitants of the household, non-Iranian nationalities or those who were not consent to the study were excluded. The demographic characteristics of this province were presented in Table 1.

**Table 1:** Demographic data of the study population.

	**Zahedan **	**Khash **	**Saravan**	**Iranshahr **	**Nikshahr **
**Subjects interviewed**	1615	469	630	648	375
**Samples collected**	1551	353	438	593	369
**Male**	48%	43%	44%	48%	45%
**Age**	29±0.4	27±0.7	28±0.6	28±0.6	28±0.8
**Rural**	15%	66%	59%	57%	92%
**Currently married**	55%	55%	57%	64%	63%
**History of blood transfusion**	5.2%	4.1%	3%	5.6%	2.4%
**Addiction**	2.2%	1.9%	4%	6%	5.9%
**IV addiction**	0.1%	0.0%	0.0%	0.2%	0.0%
**Tattoo**	13.3%	9.4%	9.2%	16%	0.5%
****	Chabahar	Kenarak	Total		
**Subjects interviewed**	619	170	4526		
**Samples collected**	564	117	3989		
**Male**	42%	50%	46%		
**Age**	29±0.6	28±1.5	28±0.2		
**Rural**	66%	58%	48%		
**Currently married**	68%	56%	59%		
**History of blood transfusion**	2.7%	3.5%	4.2%		
**Addiction**	4.2%	2.4%	3.5%		
**IV addiction**	0.2%	0.0%	0.1%		
**Tattoo**	1.5%	1.8%	9.6%		

Our sample size (n=4536) by using [DEFF*Np(1-p)]/ [(d^2^/Z^2^_1-α/2_*(N-1)+p*(1-p)] equation, when N was population size (1,440,518), P was hypothesized as % of frequency of outcome factor in the population (5%), d was confidence limits as % of 100 (absolute +/- %, here 2%) and DEFF was design effect for cluster surveys that was set to 1 had 99.99% confidence level.

Clustered random sampling was used. One hundred clusters were selected from each district within province with a cluster size of 10. Postal code or family registry code was used to randomly select the first household for each cluster. Blood samples were obtained from each subject and a questionnaire was completed by a trained interviewer. The questionnaire included demographic and anthropometric data and risk factors for hepatitis.

The blood samples were transferred to the regional laboratory. After separation of serum from blood samples in local laboratory, serums were frozen in -20°C and transferred to the central laboratory of Iranian Blood Transfusion Organization. Positive HBsAg cases were checked for anti-HDV antibody. HBsAg, HBcAb and anti-HDV antibody were evaluated using Enzygnost HBsAg, 5.0 kit (Dade Behring, Germany), Hepanostica anti-HBc Uni-Form kit (Biomerieux, France), and DiaSorin ELISA kit (Italy), respectively.

The study was approved by the Institutional Review Board of the Baqiyatallah University of Medical Sciences, Baqiyatallah Research Center for Gastroenterology and Liver Disease and Iranian Blood Transfusion Organization. Written informed consent was obtained from all subjects before data collection.

Statistics of all variables were summarized in tables. Continuous variables were presented as mean values±standard deviation (SD), while qualitative and discrete variables were presented as absolute and relative frequencies in the form of percentage. Chi-Square test was applied to assess associations between categorical variables. The 95% CI for rate of HBcAb or HBsAg positivity was calculated by Mid P-Exact method if NPQ = 5 when N was sample size, P was the rate of positive cases and Q was 1 – (rate of positive cases). If NPQ was = 5, then normal approximation method (Wald) was used to make 95% CI. The comparison between continuous and qualitative variables was performed by student's t-test. Multivariable logistic-regression analysis involving recorded risk factors and patients’ characteristics that had significant p value in univariate analysis plus HBV serostatus was performed to identify independent predictors of positive HBV seromarkers. A stepwise procedure was deployed with p=0.05 as the threshold level for variables to be entered into and retained in the final model, and p=0.1 as the threshold level for variables to be removed. All computations were carried out using SPSS software (Version 18, Chicago, IL, USA).

## Results

**Fig. 1:**
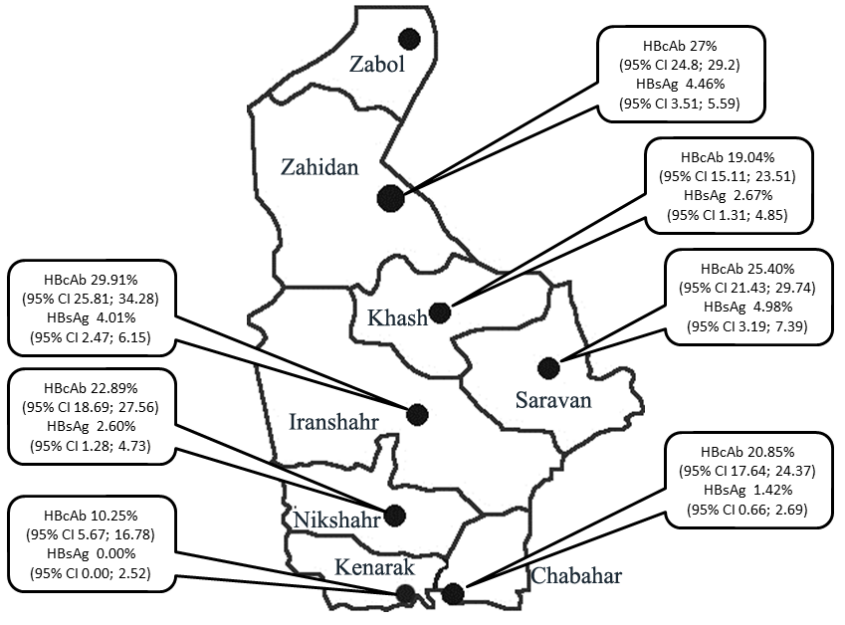
Geographical distribution of HBcAb and HBsAg seropositivity in various districts of Sistan and Baluchistan Province.

A total of 4526 subjects were interviewed from 7 districts of Sistan and Baluchistan Province. Zabol district was excluded from the study due to lack of cooperation and local difficulties and 537 subjects that refused to give blood samples or had inadequate samples or with missed laboratory data were excluded. A total of 3989 participants were analyzed. The demographic characteristics of study population were shown in Table 2. Totally, 941; 23.60% (95% CI 22.30; 24.93) and 135; 3.40% (95% CI 2.85; 3.98) subjects from 3989 participants were HBcAb and HBsAg positive respectively. In addition, we found 8 cases of positive anti-HDV antibody. Figure 1 shows the rate of positive HBV seromarkers in various districts of Sistan and Baluchistan Province. Kenarak was the only district without any detected cases of both HBsAg despite HBcAb prevalence of 10%. Iranshahr had the highest prevalence of HBcAb (29%) and Kenarak had the lowest (10%). HBsAg ranged between 0 to 5%. In univariate analysis, there was a significant heterogeneity in the rate of HBsAg (p=0.003) and HBcAb (p=0.003) among various districts. The age-specific seroprevalence of HBcAb for each sex was shown in Table 2. It can be seen that HBcAb seroprevalence and the differences between males and females increased with age group.

**Table 2:** Seroprevalence of HBcAb in male and female subjects in different age groups.

**Age group**	**Male (%)**	**Female (%)**	**Total (%)**
**6–29 years**	13	13.8	13.5
**30–45 years**	26.2	25.7	26.3
**46–65 years**	54	38	45.6

**Fig. 2:**
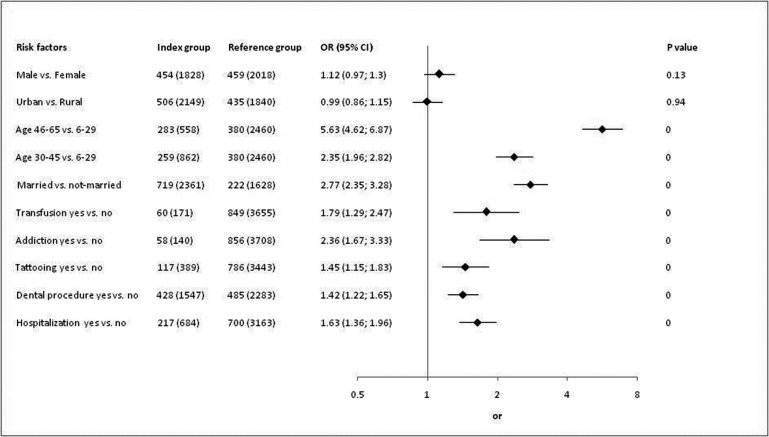
Univariate analysis.

**Fig. 3:**
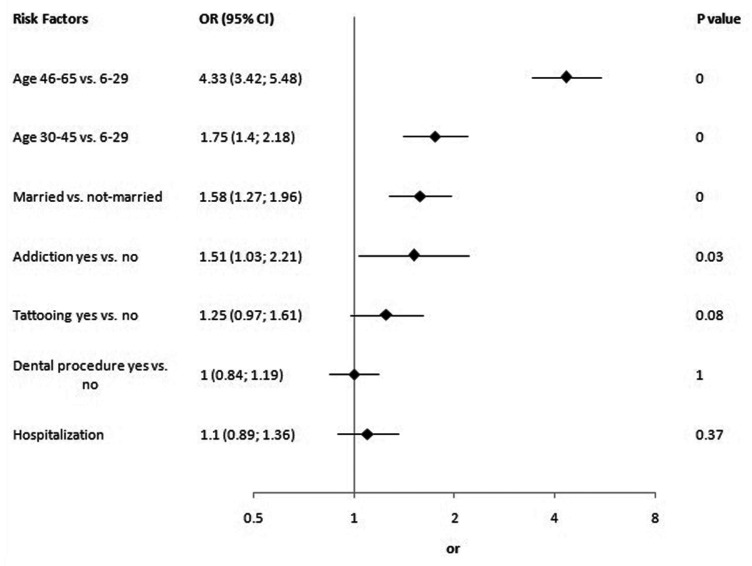
Multivariate analysis.

Figure 2 shows univariate analyses. Age, marital status, transfusion, addiction, tattooing, history of dental procedures and hospitalization had significant p value in univariate analyses but only age, marital status and addiction were significant risk factors of HBsAg or HBeAb positivity in multivariate analysis (Figure 3).

## Discussion

Our study indicates that seroepidemiology of HBV infection in general population of Sistan and Baluchistan Province was higher than other parts of the country.[7][8][10][12] Based on our survey, it can be estimated that 8,131 chronic HBsAg carriers are living in this province and can be reservoir for transmission of HBV infection through close contacts, sharing needles or nosocomial interventions. Furthermore, these patients are at great risk of hepatocellular carcinoma, cirrhosis and liver failure that their management would pose a heavy burden on the local public health system. In addition, 56,727 individuals who had positive HBcAb are a threat to the public health, since a proportion of them could have occult HBV infection with very low level of HBV-DNA in the serum and liver tissue but be negative for serum HBsAg.[19] Patients with occult HBV infection can transmit the infection and have higher incidence of liver cirrhosis and hepatocellular carcinoma.[20] Unfortunately, there were no prior data available from this province to help to monitor changing epidemiology of HBV through time.

In multivariate analysis age, addiction, and marital status were independent risk factors for HBV seropositivity. Older subjects and married individuals had higher probability of HBV seropositivity. Age is a common risk factor that almost is reported in all of seroepidemiologic studies of HBV infection.[12][21][22] The reason is that the risk and cumulative frequency of high risk behaviors increase with age and consequently increase the likelihood of HBV infection. In addition, mandatory HBV vaccination for neonates began in 1993, therefore those who had been born before this year had not received HBV vaccination and had a higher risk of infection. Although, sex was not a risk factor but as it was shown in Table 2, in age group 46-65 years, males had significantly higher rate of HBV seropositivity. Moreover our analysis suggests that nosocomial HBV transmission does not play important role in this high seroepidemiology of HBV infection. However, it seems that age, addiction and intrafamilial transmission were major determinants of HBV infection in general population of Sistan and Baluchistan Province. Respectively, 36 and 27% of addicted and married subjects were HBcAb positive. Therefore, checking couples before marriage and vaccination are advisable since they can decrease the prevalence of HBV infection in this province considerably.[23] Furthermore, effectiveness of HBV vaccination of individuals with high risk behaviors such as prisoners, sex workers, in addition to free disposable syringe for the illegal intravenous drug abusers was reported before.[24]

The rate of HBV infection in Sistan and Baluchistan Province was significantly higher than other parts of Iran. Approximately 25% of general population in this province had previous exposure to HBV and 3% were HBsAg carriers. Intrafamilial and Addiction were major routes of HBV transmission in this province.
